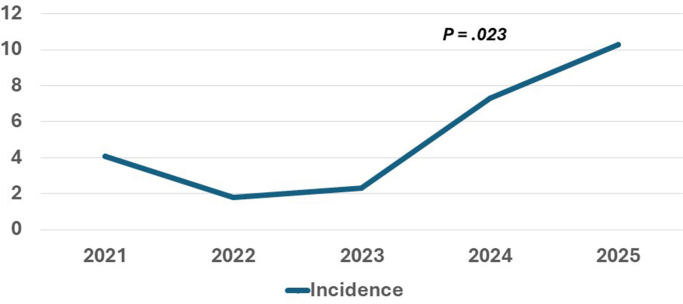# Hepatocellular Cancer in the Real World—Rising Detection With Same Outcomes

**DOI:** 10.1016/j.gastha.2026.100913

**Published:** 2026-03-03

**Authors:** Ali S. Taha, Lana Stevenson, Caroline McCloskey, Wilson J. Angerson

**Affiliations:** 1 Department of Gastroenterology, University Hospital Crosshouse, Kilmarnock, Scotland, UK; 2 Medical School, University of Glasgow, Glasgow, Scotland, UK

Hepatocellular cancer (HCC) is the world’s third commonest cause of cancer-related death. HCC surveillance with 6-monthly liver ultrasound scans is recommended to facilitate early tumor detection, plan curative intervention, where possible, and improve survival.^1^^,^^2^ For HCC surveillance to be effective, a number of actions are required including recognition of patients who are at risk, clinicians arranging screening tests, and patients engaging in the process with the intention to complete the surveillance and undergo other consequential interventions on a timely schedule.^2^, ^3^, ^4^ More recently, the use of both ultrasound scans and alpha-fetoprotein (AFP) has been recommended in order to improve the yield of HCC surveillance.^5^ The impact of this strategy on the detection rates and the overall survival of HCC patients in the real world are not clear.

In this article, we report our experience in the use of both ultrasound and AFP for surveillance and in targeting patients at risk of HCC by seeing them at dedicated cirrhosis clinics. We demonstrate that the incidence of HCC has risen as a result. However, this rise has not been matched by improvement in the overall survival rates. Therefore, the criteria for selecting patients for screening might need to be revised, and an early palliative approach needs to be considered, as appropriate.^6^

This is an observational analysis of prospectively collected data on patients diagnosed with HCC at our center, affiliated to University of Glasgow, and serving a population of 220,000 located in Southwest Scotland, over a 5-year period, 2021–2025. In 2021 and 2022, patients with cirrhosis, regardless of its cause, were screened for HCC while being consulted at general gastroenterology and hepatology clinics. In 2023–2025, patients with cirrhosis were screened while attending dedicated cirrhosis clinics.

As mentioned previously, our HCC surveillance was based on both ultrasound and AFP measurement. HCC, initially suspected on ultrasound scans and/or abnormal AFP level, was investigated further with triphasic computed tomography scans and magnetic resonance imaging scans. In some cases, such imaging modalities had to be repeated for diagnostic clarification, together with further computed tomography scans of the chest, abdomen, and pelvis for staging. Liver biopsies were also carried out in some patients, particularly if the radiological tests were still uncertain about the diagnosis. The diagnostic tests, their repeats, and the appropriate therapeutic interventions were completed as advised by our regional HCC multidisciplinary team comprising hepatologists, liver pathologists, diagnostic and therapeutic radiologists, surgeons, and oncologists.

Patients’ details were recorded including their demography, performance status as measured by the Eastern Co-operative Oncology Group (ECOG) score, background liver diagnosis, Child-Pugh score, Barcelona Clinic Liver Cancer (BCLC) stage, type of intervention given for their HCC, if any, and their survival after HCC diagnosis.

Statistical methods included an exact incidence ratio test, the Mann-Whitney test, and Fisher exact test, as appropriate. They also included the Kaplan-Meier survival curves with the log-rank test and Cox regression for survival analysis.

The incidence of HCC in our region has risen from 4.1 in 2021 to 10.3 per 100,000 population per annum in 2025 (*P* = .023), as shown in Supplementary Figure 1. Detection rates were greater in dedicated cirrhosis clinics (2023–2025) vs general hepatology clinics (2021–2022) (detection rate ratio of 3.14; 95% confidence interval [CI] [1.77–5.58]; *P* < .0001). Details of HCC patients are shown in Table. Of all 51 HCC cases detected, 27 (53%) had metabolic dysfunction-associated steatotic liver disease, 14 (27%) had alcohol-related liver disease, and 10 (20%) had other liver conditions. Nineteen patients (37%) were suitable for interventions (chemoembolization, ablation, transplant, or chemotherapy), while 32 (63%) had no intervention: observation was recommended for 17 (33%) and palliation for 15 (29%). Despite the higher detection rates, there was no detectable difference in survival rates (Kaplan-Meier analysis with a log-rank test, *P* = .8). All deaths (n = 21; 41%) occurred within 12 months of diagnosis. Cox regression analysis with a forward stepwise selection procedure to identify those variables independently associated with survival showed that the BCLC stage had the strongest association (odds ratio for 1-unit increase, 2.34; 95% CI [1.54–3.57]; *P* < .001), followed by ECOG score (odds ratio 1.51; 95% CI [1.01–2.31]; *P* = .044).

**Table:** Demographic and Other Details of HCC Patients Identified in Dedicated Cirrhosis vs General Hepatology Clinics

	General clinics (2021–2023), n = 18	Dedicated clinics (2024–2025), n = 33	All clinics, N = 51
Age, y^a^	61 (54–74)	67 (62–73)	66 (59–73)
Males	11 (61%)	19 (58%)	30 (59%)
AFP^a^	12 (5–391)	7 (4–16)	8 (4–25)
Child-Pugh score^a^	5 (5–7)	5 (5–6)	5 (5–6)
ECOG score^a^	1 (0–1)	1 (0–1)	1 (0–1)
Diagnosis			
MASLD	8 (44%)	19 (58%)	27 (53%)
Alcohol liver disease	5 (28%)	9 (27%)	14 (27%)
Hepatitis C	1 (6%)	1 (3%)	2 (4%)
Autoimmune hepatitis	2 (11%)	1 (3%)	3 (6%)
Hemochromatosis	0 (0%)	3 (9%)	3 (6%)
Sclerosing cholangitis	2 (11%)	0 (0%)	2 (4%)
BCLC stage			
0	4 (22%)	9 (27%)	13 (25%)
A	1 (6%)	3 (9%)	4 (8%)
B	4 (22%)	10 (30%)	14 (27%)
C	1 (6%)	7 (21%)	8 (16%)
D	8 (44%)	4 (12%)	12 (24%)
Intervention			
None (please see below)	11 (61%)	21 (64%)	32 (63%)
TACE	3 (17%)	4 (12%)	7 (14%)
TACE and liver transplantation	1 (6%)	1 (3%)	2 (4%)
Ablation	2 (11%)	1 (3%)	3 (6%)
TACE and ablation	0 (0%)	3 (9%)	3 (6%)
Systemic anticancer therapy	1 (6%)	3 (9%)	4 (8%)
No intervention			
Observation	5 (28%)	12 (36%)	17 (33.3%)
Palliation	6 (33%)	9 (27%)	15 (29.4%)

MASLD, metabolic dysfunction-associated steatotic liver disease; TACE, transarterial chemoembolization.

a Median (interquartile range).

World incidence of HCC has been rising.^1^, ^2^, ^3^, ^4^, ^5^, ^6^ The Americas, Northern Europe, and Oceania generally have HCC incidence rates below 5 per 100,000.^7^ This explains our HCC incidence of 4.1 in 2021. The steady rise we observed in the following years, more than doubling to 10.3 in 2025, probably resulted from our targeting of patients at risk and using both ultrasound and AFP for surveillance. It is also interesting to see that metabolic dysfunction-associated steatotic liver disease was the main diagnosis in patients with HCC, in line with the findings of other works.^8^^,^^9^

Our rising incidence was not matched by improvement in the overall survival of HCC patients, possibly because the rise in BCLC stage 0 (22%–27%) and the drop in BCLC stage D (44%–12%) were not statistically significant, nor were the changes in ECOG performance scores. Cox regression analysis showed both BCLC stage and ECOG scores to be independent variables in predicting survival of HCC patients. Such variables had impacted the decision of the multidisciplinary team to palliate some cases or to observe others in the hope of becoming more suitable for therapeutic interventions.

It could be argued that there is no rationale for surveillance in patients who would not be candidates for anticancer treatment if HCC was diagnosed.^6^ In the real world, it might not be easy to identify such patients or to decide on the most appropriate intervention once HCC is diagnosed and hence the need to involve a multidisciplinary team and for these aspects of care to be clarified further.

Our work is limited by being single-center and by the relatively small numbers of HCC cases studied. Despite these, our incidence is in line with that predicted by international epidemiological data, and it had risen further with targeting patients at risk and with the use of both ultrasound and AFP for surveillance. Our interventions were based on the advice of a dedicated multidisciplinary team. We have also demonstrated the key roles of BCLC and performance status in impacting survival of HCC patients.

## Figures and Tables

**Supplementary Figure 1:**